# P-938. Percutaneous Mechanical Aspiration in Drug Use Associated-Infective Endocarditis

**DOI:** 10.1093/ofid/ofae631.1129

**Published:** 2025-01-29

**Authors:** Jessica Metlay, Nicholas Blair, Matt Scherer, Kartik Kodali, Sanjum Sethi, Sahil A Parikh, Robert Zilinyi, Magdalena E Sobieszczyk

**Affiliations:** Columbia University Medical Center, New York, New York; Columbia University Medical Center, New York, New York; Columbia University Irving Medical Center, New York, NY; Columbia University Medical Center, New York, New York; Columbia University Medical Center, New York, New York; Columbia University Irving Medical Center, New York, NY; Columbia University Medical Center, New York, New York; Division of Infectious Diseases, Department of Medicine, Vagelos College of Physicians and Surgeons, New York-Presbyterian Columbia University Irving Medical Center, New York, NY, USA, New York, New York

## Abstract

**Background:**

Intravenous drug use is a known risk factor for infective endocarditis (IE). Incidence of drug use associated-infective endocarditis (DUA-IE) has increased in the US during the opioid epidemic. Management of DUA-IE, especially in patients deemed high surgical risk, is an area of need. Angiovac (AngioDynamics, Latham, NY) is a percutaneous mechanical aspiration device used for high-risk surgical patients to debulk right-sided intravascular material, including in IE, using a venous drainage cannula and extracorporeal veno-venous bypass. We review characteristics and outcomes of Angiovac for DUA-IE

**Figure:**
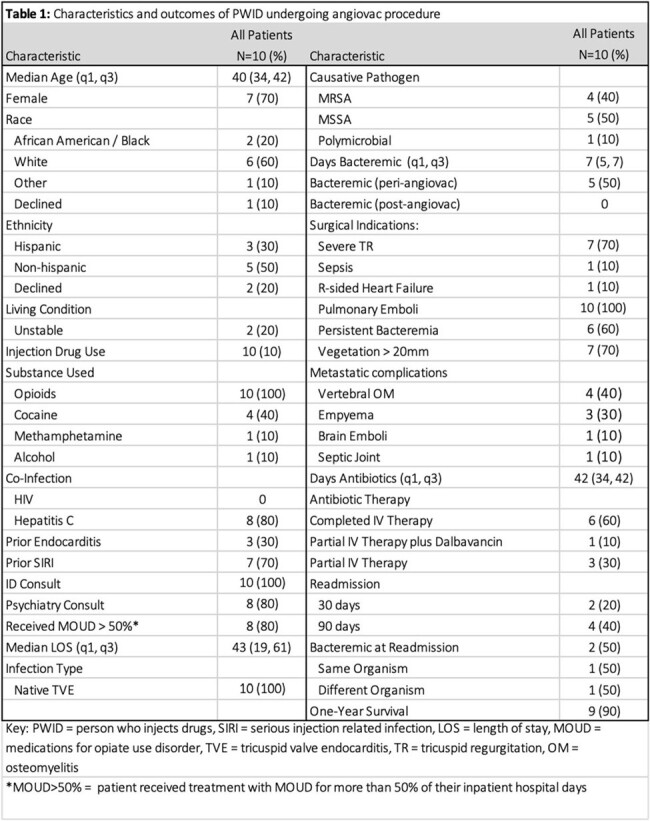

**Methods:**

Descriptive analysis of Angiovac cases performed between 2019-2023 at a large academic medical center in northern Manhattan. Cases were included if there were concurrent diagnoses of IE and intravenous drug use

**Results:**

Ten cases were identified; all involved the native tricuspid valve. Overall, median age was 40 years, 70% (n=7) were female, 60% white and 80% Hepatitis C infected. Median duration of bacteremia was 7 days; 9 (90%) patients had *Staphylococcus aureus*. Seven patients had severe tricuspid regurgitation and 7 had at least one metastatic complication, most commonly vertebral osteomyelitis (4, 57%). Five patients remained bacteremic at time of Angiovac. Post-procedure cultures cleared in 1-7 days (median 3 days). Median duration of antibiotics was 42 days including use of intravenous (including dalbavancin) or oral. Most patients received medications for opioid use disorder while hospitalized. 40% (n=4) were readmitted by 90 days and two re-hospitalized patients had bacteremia; one of these cases was polymicrobial including the previous organism (methicillin-susceptible *staphylococcus aureus*). Nine patients were alive one-year post-procedure. The patient with polymicrobial bacteremia died after cardiac arrest in the context of suspected ongoing drug use

**Conclusion:**

Angiovac led to blood culture clearance and 90% one-year survival in a small cohort of patients with DUA-IE. Multidisciplinary approaches for care of these patients are critical and this minimally invasive procedure can be an important part of management. Future studies are needed to identify patients who would benefit from Angiovac and define optimal course of antibiotics post-procedure

**Disclosures:**

**Kartik Kodali, n/a**, AbbVie: Stocks/Bonds (Public Company) **Sanjum Sethi, MD**, AngioDynamics: Advisor/Consultant|Chiesi: Honoraria|Inari: Advisor/Consultant|Janssen: Advisor/Consultant|Penumbra: Advisor/Consultant **Sahil A. Parikh, MD**, Abbott: Advisor/Consultant|Abbott: Grant/Research Support|Advanced Nanotherapies: Ownership Interest|Boston Scientific: Advisor/Consultant|Boston Scientific: Grant/Research Support|Cordis: Advisor/Consultant|Encompass Vascular: Ownership Interest|Inari: Advisor/Consultant|Medtronic: Advisor/Consultant|Penumbra: Advisor/Consultant|Penumbra: Grant/Research Support|Philips: Advisor/Consultant|Reflow Medical: Grant/Research Support|Shockwave Medical: Advisor/Consultant|Shockwave Medical: Grant/Research Support|Surmodics: Grant/Research Support|Terumo: Advisor/Consultant|TriReme Medical: Grant/Research Support|Veryan Medical: Grant/Research Support

